# Normality and compassionate care: experiences from advanced cancer patients in their last time at home

**DOI:** 10.1186/s12875-024-02499-x

**Published:** 2024-07-06

**Authors:** Bardo Driller, Carolin Maienborn, Elin Margrethe Aasen, Adriana Kolstrøm, Bente Talseth-Palmer, Torstein Hole, Kjell Erik Strømskag, Anne-Tove Brenne

**Affiliations:** 1 Department of Oncology, Møre and Romsdal Hospital Trust, Ålesund, Norway; 2 Department for Research and Innovation, Møre and Romsdal Hospital Trust, Ålesund, Norway; 3 Department of Clinical and Molecular Medicine, Faculty of Medicine and Health Sciences, Norwegian University of Science and Technology, Trondheim, Norway; 4 School of natural sciences, Trinity College Dublin, University of Dublin, Dublin, Ireland; 5 Department of Health Sciences Ålesund, Faculty of Medicine and Health Sciences, Norwegian University of Science and Technology, Ålesund, Norway; 6 Home care department, Kristiansund municipality, Kristiansund N, Norway; 7 School of Biomedical Sciences and Pharmacy, College of Health, Medicine and Wellbeing, University of Newcastle, Newcastle, Australia; 8 NSW Health Pathology, Newcastle, NSW Australia; 9 Department of Surgery and Emergency Medicine Molde Hospital, Møre and Romsdal Hospital Trust, Molde, Norway; 10 Department of Circulation and Medical Imaging, Faculty of Medicine and Health Science, Norwegian University of Science and Technology, Trondheim, Norway; 11 St. Olav’s Hospital, Cancer Clinic, Trondheim University Hospital, Trondheim, Norway

**Keywords:** Advance Care Planning, Cancer, Home Care, Palliative Care, Patients perceptions, Primary Healthcare

## Abstract

**Background:**

Many cancer patients prefer to receive palliative treatment at home, as it allows them to be in a familiar and comfortable environment. Integrating Advance Care Planning (ACP) into routine practice in primary healthcare helps patients and their relatives prepare for end-of-life (EoL) care in accordance with patients’ preferences. This includes the option to spend their final days at home if desired. The aim of this study was to gain insights from experiences of advanced seriously ill cancer patients at home while receiving palliative treatment and being engaged in ACP within primary healthcare settings.

**Method:**

This study employed a qualitative design, utilizing individual, semi-structured interviews that were analysed through reflexive thematic analysis, employing an abductive approach with a latent-level focus. The study included interviews with 12 participants with cancer who were receiving palliative care, had an estimated lifetime under 3 months, and had undergone an organized ACP approach in primary healthcare, documented with a palliative plan.

**Results:**

Participants emphasized the importance of (1) *Preserving normality at home*, maintaining a sense of routine, comfort, and familiarity in the face of present and future challenges. The top obstacles for success identified by participants included (1a) *The challenge of deterioration* and the dual aspects of (1b) *The value and burden of family caregivers*. Cancer treatment placed a significant demand on patients due to side effects. Family caregivers played a crucial role for participants, providing support in daily life and serving as a key factor in the overall decision to which extend they are able to involve in support and care at home in the future. (2) *Compassionate health care personnel (HCP) made a difference* by fostering a culture of understanding participants’ concerns, fears, and preferences, which was a key element that built and maintained trust for the participants. (3) *Preparing for the future*, especially EoL discussions initiated by healthcare personnel, was deemed important but, at times, uncomfortable for participants as it confronted them with reality. Guidance from ACP provided them with a sense of certainty and control.

**Conclusion:**

Preserving normality at home, along with the desire to stay at home for as long as possible, is a crucial goal for advanced cancer patients. Consistent professional communication and care in primary healthcare play a key role in building and maintaining trust, as well as fostering a sense of certainty and control for the participants.

## Introduction

Many cancer patients undergoing palliative treatment express a preference to spend the majority of their remaining time in the comfort and familiarity of their own home [1, 2]. Experiences at home during an advanced stage of illness are unique and complex, varying widely depending on the level of support available and individual preferences and circumstances [3]. Family members or caregivers often play a significant role in providing care at home, offering a sense of security and peace that can enhance the overall well-being of patients [4]. However, balancing the demands and satisfaction of caring for someone in the advanced stages of illness at home can be challenging [5]. The involvement of family caregivers in palliative care may not always align with the magnitude of their responsibilities [6]. Home-based care for advanced cancer patients, with access to community resources and support, typically involves coordination among various healthcare professionals [7].

While there is a broader body of research exploring the experiences of healthcare personnel (HCP) and family caregivers, only a limited number of studies have examined the end-of-life (EoL) experience of patients in the context of home as their place of living and receiving care [8, 9]. This study aims to address this gap. Gaining more knowledge about patients’ experiences can contribute to a better understanding and empathy for individuals at the EoL, potentially assisting healthcare personnel in providing compassionate and patient-centred care.

Cancer patients undergoing palliative treatment and their families may engage in discussions about EoL planning. Advance care planning (ACP) is defined as the ability to enable individuals to define goals and preferences for future medical treatment and care, to discuss these goals and preferences with family and healthcare providers, and to record and review these preferences if appropriate [10]. ACP is an ongoing process that includes reviewing the patients´ current medical condition and prognosis [11]. ACP has evolved over time, emphasizing patient-centred care, informed decision-making, and focusing on communication rather than on specific interventions or outcome [12, 13].

Despite an increasing interest in ACP, research suggests that the uptake of ACP discussions in clinical practice is low and may occur at inappropriate times [14, 15]. This could be attributed to the fact that participation in ACP can be accompanied by unpleasant feelings, although many patients report benefits from advance care planning as well [16]. The purpose of ACP often involves preparing for incapacity and preparing for dying [17]. Patients and family members may perceive fear of discussing their relative’s EoL care and uncertainty about the remaining time as barriers to engaging in ACP [18]. This suggests a need for ACP to be personalized in a form that is both feasible and relevant at moments suitable for the individual patient [19]. Patients often prefer HCPs to initiate necessary communication, emphasizing the role of personal relationships with HCPs as part of a social process [16]. In usual practice, integrating day-to-day care planning with ACP can be challenging, and ACP documentation may be difficult to find and use [20]. Most studies focus on evaluating patients’ experiences with ACP conversations rather than the entire ACP process, and these studies often occur in the early stages of the ACP approach [21]. The entire ACP process involves documenting preferences, sharing the palliative care plan between the patient and HCP, reviewing and updating the plan, and integrating it into care planning.

## Aim

The aim of this study was to acquire insights into the experiences of advanced seriously ill cancer patients at home while undergoing palliative treatment and engaging in ACP in primary healthcare settings.

The research question guiding this study was: How do palliative cancer patients experience their last time at home?

## Method

### Study design

This study employed a qualitative design involving individual, semi-structured interviews. The data were analysed using reflexive thematic analysis, employing an abductive approach with a latent-level focus.

### Study setting

The study was carried out in a rural healthcare area in West-Norway with an approximate population of 100,000 residents. The municipalities within the catchment area maintained collaborations with two local hospitals. In addition to the assistance provided by general practitioners (GPs) and home care nurses, community cancer nurses offered additional support to both patients and family caregivers. Community cancer nurses typically served as the first contact for patients accessing community care. They played an important role in coordinating essential care services and were at the frontline of communication regarding EoL care. Cancer patients, based on their needs and symptom burden, could avail themselves of the services provided by the hospital-based palliative care (PC) team through referrals from either hospitals or primary healthcare.

Since June 2018, community cancer nurses in our region were able to provide organized ACP conversations and summarizing palliative plans to all patients with life-limiting diseases, including those with non-curable cancer. They utilized actively supportive tools such as a standardized template for the palliative plan within the electronic patient journal (EPJ), informational flyers for both healthcare providers and patients, and an information video available on the dedicated website (www.palliativplan.no).

Collaboratively with the GP, community cancer nurses decided on the timing and necessity of offering the ACP conversation to the patient, with the option of conducting it at the patient’s home. From a prior assessment of 125 ACP conversations in primary healthcare, we observed that approximately two-thirds of these discussions involved the presence of the GP, with the majority of the remaining conversations including a physician from the hospital-based PC team. 15% of the ACP conversations occurred without a physician present. In such instances, community cancer nurses obtained necessary medical information from specialist healthcare, often as a result of consultations with the hospital-based PC team during hospital admissions or visits to the cancer outpatient clinic [22]. During the conversation, patients shared their wishes, their perspective on their current health condition, and responded to questions about their healthcare priorities. The GP then confirmed the final version of the summarizing palliative plan, which could be shared with any future healthcare providers upon request. The plan was subject to reassessment and updates as needed, particularly in the case of significant changes in the patient’s medical condition [22].

### Analysis and interpretation of interview data

The analysis, conducted from March to October 2023, followed these steps [23]:

1. Get familiar with the data / generate initial codes.
2. Search for patterns, themes, or subthemes in the codes.
3. Review themes (and subthemes).
4. Refine, define, and finalize themes.
5. Produce the report.

Reliability checks were consistently performed within the researcher team throughout the analysis and interpretation process to verify the present findings and ensure the accuracy of the study.

Our research focused on understanding the subjective experiences, views, and opinions of the participants, employing reflexive thematic analysis to reveal patterns and meanings in the data. The analysis entailed constant navigation between the entire dataset, coded data extracts, and the analytical process. A reflexivity journal documented our coding and analysis process. Codes assigned to data extracts were organized into preliminary themes and sub-themes using an Excel spreadsheet, linked to their source in the transcripts.

We adopted an abductive approach, analysing data without preconceptions or a pre-existing coding frame, generating potential explanations or hypotheses based on observations. These were compared to existing theory. Literature review in the early stages of analysis was limited to maintain an open-minded approach. The latent-level approach involved interpreting and thematising meanings to theorize social and structural conditions, supporting the conclusions provided.

The findings were presented with data extracts to highlight key points, and discussions among all authors ensured a comprehensive consideration of perspectives. Themes and subthemes were refined after reviewing individual thematic coding results, elevating them to a certain abstraction level to interpret the participants’ contributions (Table 1).

**Table 1:** Illustration of analytic steps followed to identify relevant themes

Data-extract	Code	Sub-theme	Theme
What is important to me in everyday life is that I can stay at home and do things I love.	Wish and challenge to manage daily life at home themselves		Preserving normality at home
Being sick has become a habit. I had several challenges and generally it has gotten worse over time.	Dependency on wellbeing (including influence of cancer treatment)	The challenge of deterioration	Preserving normality at home
When I die, I don’t want to lie at home because I don’t want the family to have to take responsibility for me.	What to expect from and how to protect family (including preparing EoL and limits)	The value and burden of family caregivers	Preserving normality at home

### Sample and requirement

A variation sampling strategy was employed to ensure diversity in age, gender, and cancer diagnoses among male and female participants. The inclusion criteria were as follows: (1) advanced non-curable cancer in a palliative setting with an estimated survival time of less than three months, (2) residing in a defined rural region in West-Norway, where ACP conversations and palliative plans were offered in primary healthcare since 2018, (3) completion of the entire process of an organized ACP conversation with a summarizing palliative plan in primary healthcare, and (4) ability to communicate in Norwegian.

Community cancer nurses in primary healthcare informed the study physician (BD) about potential participants. The study physician confirmed eligibility, occasionally collaborating with an oncologist to estimate survival time (Table 2).

**Table 2:** Recruiting of participants

Recruiting of participants
Community cancer nurses informed about **28 patients** with an organized ACP conversation in primary health and a summarizing palliative plan
3 had too long estimated time of survival
1 was cognitive not able to join
4 died before they could be asked
**20 possible participants were asked**
3 died before they could answer
2 said no
**15 participants said yes**
2 withdrew consent because they felt too bad
1 cancelled the interview twice and died
**12 participants were interviewed**

### Data collection and interview guide

Data were collected through individual semi-structured interviews conducted between April 2021 and September 2022. It’s noteworthy that the participants were not receiving care from the interviewing study physician or nurse. The interviews aimed to explore participants’ feelings, priorities, experiences of serious illness, home care, and the dynamics of their everyday life. Participants shared insights into their interactions with healthcare professionals, including their experiences with ACP and palliative plans.

The interview guide, developed by two authors (BD, AK), drew on their prior knowledge of the topic and focused on participants’ key experiences. Adjustments were made to the guide after the initial two interviews, giving more consideration to the relationships with healthcare personnel and family dynamics. The guide initiated with open-ended questions, allowing flexibility in the order of additional questions. This approach facilitated a comprehensive exploration of patient experiences and enabled the interviewer to delve into new and significant topics raised by the participants.

The interviews were conducted by two authors (BD, AK) in either the patient’s home (11 interviews) or the hospital (1 interview). For the first two interviews, both authors participated, working together — one as the interviewer and the other as an observer, providing feedback to the interviewer afterward. Subsequently, BD conducted nine interviews alone, while AK conducted one interview alone. In three instances, the next of kin were present during the interview but did not actively participate in the conversation.

Digital audio recordings were used to collect data, and the recordings ranged in duration from 18 to 41 min, with an average length of 28 min. In one case, a participant used a data tablet for communication assistance, with the interviewer reading aloud what the patient wrote down. The inclusion of participants with diverse characteristics met the requirements for variation, involving all 12 participants. The transcriptions of the interviews were done verbatim by three authors (BD, AK, CM), and each transcription was crosschecked to ensure accuracy. Participant characteristics are detailed in Table 3.

**Table 3:** Participant characteristics (*n* = 12)

Participant characteristics (*n* = 12)
6 female / 6 male
Median age 72 years (55–81)
8/12 married or cohabitated
Cancer disease; breast [2], colon [1], prostate, pancreatic, lung, mesotheliom, kidney, leukemia, pharyngeal
9 ongoing cancer treatment, 3 treatment break or recently finished
First palliative plan median 36 weeks before interview (9–163)
Died median 11 weeks after interview (3–50)
4 died at home, 5 in nursing home, 3 in hospital

### Ethical considerations

The study was performed in accordance with the guidelines and regulations outlined in the Declaration of Helsinki and received approval from the Regional Committee for Medical and Health Research Ethics (REK; ID 168,328) and the Cancer Department at Møre and Romsdal Hospital Trust. Participants were given both verbal and written information about the study, and informed consent was obtained from all participants before their involvement. To address potential palliative needs following the interview, the interviewer ensured that participants had access to contact the community cancer nurse and provided them with the contact information for the local hospital-based PC team.

## Findings / results

The analysis resulted in three themes and two subthemes:

- Preserving normality at home [24].
  - The challenge of deterioration (1a).
  - The value and burden of family caregivers (1b).
- Compassionate HCPs make a difference [1].
- Preparing for the future (EoL) [2].

### Preserving normality at home (1)

All 12 participants expressed a strong desire to remain at home, emphasizing the importance of maintaining routine, comfort, and familiarity amidst the challenges posed by serious illness. They considered home a familiar and fulfilling environment, providing happiness and a sense of normality. Participants valued the control over their daily routines and the ability to savour simple pleasures. Emotional support and assistance from family and social connections played a crucial role, contributing to a sense of belonging.

One participant stated, “It is important for me to be together with my family and to live as normally as possible, to be with my loved ones” (P11-177). Participants viewed home as a place that boosted their self-confidence, supporting their wish for independence. This positive sentiment highlighted the significance of routine, mastery, and autonomy. However, participants acknowledged their limitations in engaging in daily activities, primarily within their physical capabilities. Home was seen as a private space with the potential for recovery from suffering. When admitted to hospital, participants appreciated to come home again to promote their overall well-being and recreation.

Expressing a preference for independence, a participant mentioned, “I prefer to be independent, prefer to manage myself, prefer to be at home. I am happy to be home” (P3-256). Participants’ sense of normality was notably influenced by the progression of their illness and the symptoms they experienced. Concerns about burdening family caregivers also played a role. One participant remarked, “It’s perfectly fine to be at home, at least when I’m as healthy as I am now, it’s worse when I’m unwell. I feel a little safer that someone from the family is around me, especially when I’m really unwell. We have found good solutions for that” (P8-42).

### The challenge of deterioration (1a)

Participants encountered challenges in regaining or maintaining control over their declining physical well-being, particularly due to side effects from cancer treatment. Despite the difficulties, the strong hope for recovery after treatment and the desire for more time to live played a significant role in sustaining their resilience through suffering. One participant shared, “Two weeks ago I was just crying and crying, there are so many side effects of the medicine that they drive you crazy. But it goes well and in the end it’s amazing what can be achieved” (P3-264).

The participants underwent a process of learning to manage their symptoms and adapt to physical limitations, aiming to maintain as much independence as possible. Despite the challenges of deterioration, they sought to maintain a positive outlook and focused on aspects within their control, such as engaging in activities or managing their nutrition. Over time, participants often developed a more adaptive approach to dealing with deterioration. One participant expressed, “Being sick has become a habit. I had several challenges and generally it has gotten worse over time” (P6-28).

However, as deterioration continued, participants faced the progression of their disease, raising doubts about their ability to manage future physical challenges while remaining at home. In cases of significant physical health deterioration, feelings of frustration, sadness, and a loss of self-esteem emerged. One participant reflected on the dilemma, stating, “Maybe it’s best to stay at home, but if I get too sick to be home, then I don’t want to be here. But I don’t really have the heart to move away, I enjoy myself so much, I really love my home” (P3-234).

### The value and burden of family caregivers (1b)

Participants derived significant benefits from their family caregivers, receiving emotional support and practical assistance around the clock. The presence of family caregivers not only provided comfort but also contributed to a sense of connection within the entire family. This constant support positively influenced participants’ emotional well-being and overall happiness, and they generally accepted a certain level of dependency on their family caregivers. One participant expressed their gratitude, saying, “It’s good to be home. I have a husband who is absolutely wonderful, he takes care of everything. I don’t have to think about anything at home” (P12-60).

Participants acknowledged the limitations of their family caregivers in addressing the challenges of future physical health deterioration and talked about to determine the level of involvement. They expressed their wishes and concerns especially around how long they would like to be at home in the interview situation but they rarely discussed willingness and availability to provide support with their family caregiver. However, participants expected their family caregiver to actively decide to which extend they are able to involve in support and care. One participant shared, “The wife must be involved in that, it’s not something I can decide (about being at home), it could be that she wants me gone before I want it myself, right?, before she becomes a health assistant or some other professional for me” (P7-164).

When it came to their preferred place of death, participants exhibited varying attitudes. Some had a wait-and-see approach with a desire to be at home if possible, while others had already decided to die in the hospital. Participants expressed concerns about the emotional involvement and burden they might place on their relatives as they deteriorated and approached the EoL. Some participants hesitated to be a major burden, suggesting that being at home might be the most burdensome for their family caregiver, or HCPs. One participant emphasized the importance of a positive ending, stating, “We have not yet discussed whether I will stay at home until the end of my life. I think it depends on whether there will be a nice ending, that there is no problem that would worsen the experience for the family, then it would be better to be in the hospital. The burden on those around me should not be so great” (P11-131).

### Compassionate HCPs make a difference (1)

Participants conveyed a positive perception of their HCPs, emphasizing their HCPs’ compassionate and patient-centred approach. They felt that their concerns were adequately addressed and appreciated the respectful and empathetic manner in which their HCPs interacted with them. As one participant expressed, “When you’re in a situation like that, the way people talk to you has an awful lot to say” (P3-100).

The positive interactions with HCPs had a significant impact on participants’ sense of certainty and overall well-being. The supportive communication style fostered a deep understanding and connection, contributing to improvements in both medical and emotional well-being. Community cancer nurses and home care nurses, in particular, were highlighted for their attentive listening, responsiveness to questions, and the ability to explain medical information in a way that participants reported to be compassionate and understandable. The role of GPs was described ambivalently. While some participants emphasized a positive relationship with their GP, others reported their GP to be less involved since they began cancer treatment in specialist healthcare. This was partly because participants didn’t perceive the need to initiate contact.

Consistent care and communication over time were emphasized as key elements that built and maintained trust for the participants. They appreciated the reassurance from HCPs, exemplified by one participant’s statement: “The fact that they have said that no matter what happens, we will help you in the best possible way, we will do everything we can for you to be well, that counts for a lot” (P3-156).

Participants expressed a sense of reliance on their HCPs for ongoing support. The discussion around disease progression, symptoms, and concerns about burdening family caregivers served as a foundation or starting point for addressing what participants referred to as ‘the vulnerable themes’.

### Preparing for the future (EoL) (2)

Participants expressed varied levels of engagement and recollection regarding their involvement in healthcare decision-making, particularly in the context of ACP conversations and palliative plans. While seven participants remembered having participated in such discussions, five explicitly stated they had no recollection of engaging in ACP conversations or having a palliative plan.

One participant mentioned, “I don’t remember if I had a conversation with the Cancer Coordinator about the palliative plan, but I have an open line with her, can call, talk to her whenever I want, express my wishes” (P7-106). This suggests that even in cases where participants did not recall specific ACP conversations, they felt they had ongoing access to express their wishes and concerns.

Generally, participants valued the clarity of information, sensitivity, respect, and guidance received during ACP conversations. This allowed them to focus on what mattered most to them, reducing uncertainty and providing a sense of control. For instance, one participant expressed, “Thanks to the fact that there is a system of cancer nurses and GPs who actually take care of a certain amount of preparation, I feel looked after in that area (time to come)” (P11-156).

However, participants also experienced a range of complex emotions and considerations during the ACP process, influenced by the depth of the discussions. Preparing for EoL was acknowledged as a challenging and emotionally taxing process. Some participants were not ready to plan for EoL, while others actively avoided contemplating their own mortality. This denial may have posed challenges for healthcare personnel in engaging participants in EoL planning.

Those willing to discuss EoL appreciated knowing that their values and preferences would be respected, providing a sense of preparedness for potential medical challenges at the EoL. In cases where relatives were present during ACP conversations, participants found comfort in the awareness that their loved ones were informed about their preferences.

Reflecting on the future, one participant expressed, “It is difficult to know how to prepare (for what happens in the future), what to expect. I’m probably not well prepared, but I think I’ve prepared myself as well as I can, also in relation to family” (P8-165). This highlights the complexities and varied emotional responses participants had toward EoL planning.

### Explanation model

**Fig. 1:**
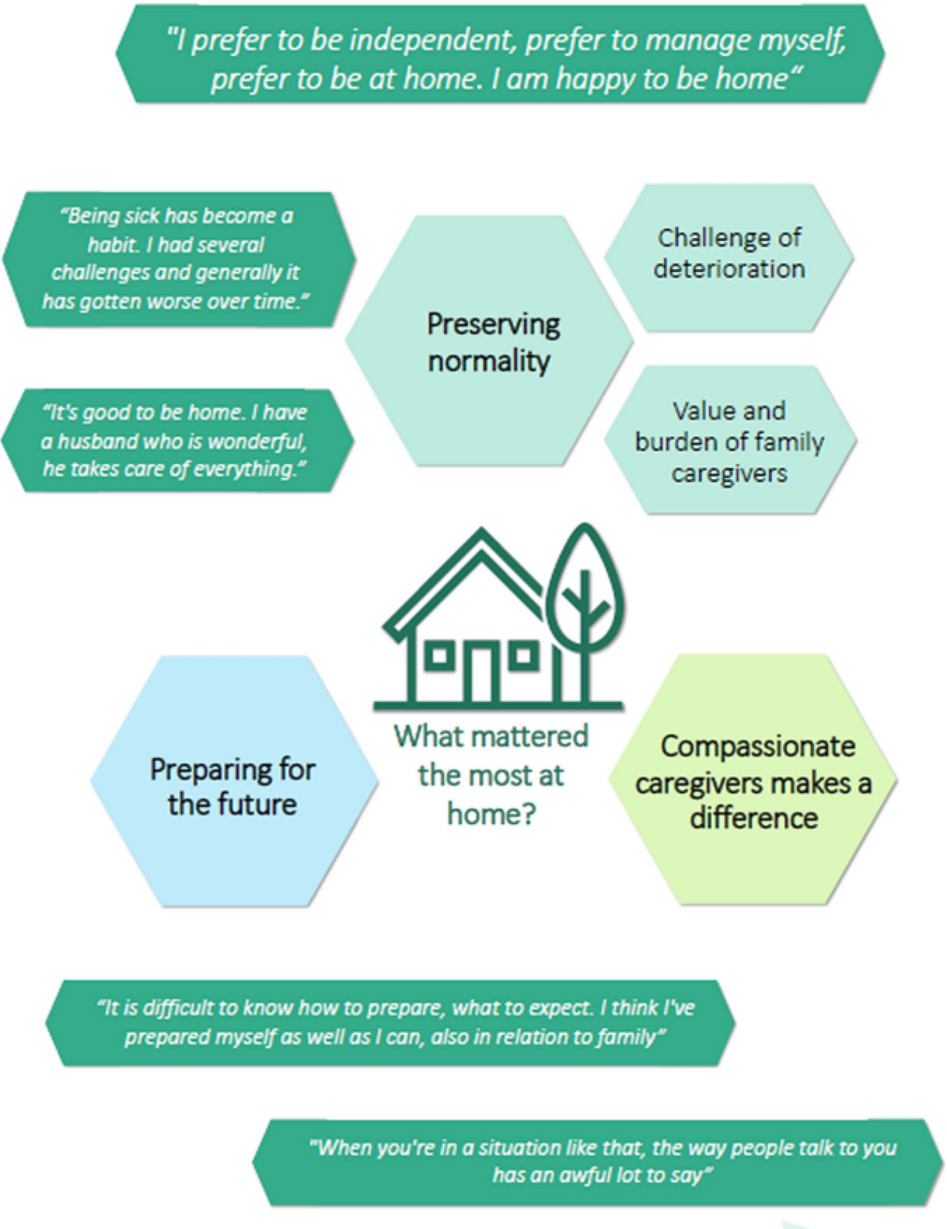
Explanation model about what mattered the most at home

The subsequent model of explanation functions as a framework for structuring and comprehending the qualitative data. Participants, while at home, employ problem-solving strategies to maintain a sense of normality in their lives. Family caregivers play an important role in this sense of normality, offering support to facilitate its preservation. The primary concerns voiced by participants revolve around the progressive deterioration of their condition and the potential burden it imposes on their family caregivers. Compassionate care provided by HCPs contributes significantly to emotional coping mechanisms, avoidance of problems, and a sense of being acknowledged and understood. Participants express appreciation for guidance received in preparing for the future, yet concurrently grapple with negative emotions associated with contemplating EoL preparations. Figure 1 gives an overview of the explanation model about what mattered the most at home.

## Discussion

In light of the rich and nuanced data of advanced cancer patients’ experiences at home, the discussion reflects on the identified themes and explores their implications for improving the quality of care, shaping future research directions, and adapting healthcare practices to meet the individual needs of those facing advanced cancer in a home setting.

All 12 participants in this study strongly expressed their desire to remain at home as long as possible and manage their lives in a familiar environment. They found fulfilment and happiness and underscored the importance of normality.

The challenge for cancer patients to maintain a sense of normality at home varies based on the cancer stage, treatment specifics, and individual circumstances [25]. Maersk et al. [3] highlighted the importance of supporting cancer patients’ identity by allowing them to engage in daily activities at home. Home and objects within it functioned as significant references to the patients’ sense of self. The desire to live as ordinary people may sometimes be a facet that patients hide from their families [26].

The participants in this study were keenly aware of the challenges associated with declining physical well-being, particularly in response to side effects of cancer treatment.

Managing side effects at home became a crucial issue, especially as cancer patients receiving chemotherapy spent more time in their home environment [27]. Koshy et al. [28] emphasized the need for supportive education and training for patients and caregivers, pointing to a negative correlation between self-care behaviours and side effects. While side effect management has evolved over time due to less toxic regimens and improvements in supportive care, patients now grapple with non-physical concerns such as effects on family and the future [29].

Being partially dependent on family caregivers was accepted to a certain degree by the participants in this study, as long as it didn’t impose too much care burden. They expected their family caregivers to play an active role in deciding and communicating the extent of care and support they were comfortable providing. However, discussing these expectations with loved ones proved challenging for the participants. This issue was addressed by 10 participants, with or without presence of the family caregiver during the interview. Participants often had a wait-and-see attitude with a preference to be at home and to die at home if possible. Some had already made their decision to prefer to die in the hospital.

Pottle et al. [5] found that the home environment facilitated a sense of normality, control, and individualized care, contributing to what family carers perceived as a “good death”. Interactions with loved ones were identified as strong support for cancer patients in coping with their condition [30]. Robinson et al. [31] observed challenges among family caregivers in identifying and making decisions about caregiving responsibilities. While patients and family caregivers often value and expect family involvement, explicit agreement on which party should take decisional leadership and who should play a supporting role remains limited [32]. End-stage palliative patients often perceive institutional care as a means to protect their families from the social, emotional, and relational burdens of dying [33]. Healthcare workers’ guidance for both patients and family caregivers can effectively identify information and support needs, utilizing evidence-based strategies to address these needs [34].

This study provides valuable insights and understanding of the interaction between HCPs and patients in primary healthcare in a home setting, as perceived by end-stage cancer patients. They felt that their concerns were adequately addressed and appreciated the compassionate and patient-centred approach of HCPs. Interestingly, for almost half of the participants, the ACP process is primarily prominent through their relationship with HCPs rather than formal ACP conversations or written documents like a palliative plan. Once confirmed, accepted, and familiar to the patient, the plan aids patients in gaining a certain amount of control in preparing for incapacity and EoL challenges.

Compassion reveals various dimensions, underscoring the significance of incorporating patient perspectives to enhance the delivery of compassionate healthcare [35]. The partnership in nursing care significantly influences cancer patients’ perceptions of quality care [36]. Essential elements in caring for patients with cancer include facilitating behaviours such as empathy, touch, comforting, and support [37]. Many cancer patients assume that HCPs will take the initiative in discussions about EoL care preferences, while HCPs may be hesitant to broach these sensitive topics [38]. Poveda-Moral et al. [18] found in an umbrella review that main barriers reported by professionals were lack of knowledge and skills to carry out ACP, a certain fear of starting conversations about ACP, and a lack of time for discussions. Patients and family members considered that the main barriers were fear of discussing their relative’s EoL, lack of ability to carry out ACP, and not knowing who was responsible for initiating conversations about ACP.

Despite potential unpleasant feelings associated with participation in ACP discussions, many patients reported benefits [39]. Johnson et al. [40] found that the complex social and emotional environments surrounding EoL planning are not adequately integrated into standardized ACP. There is a recognized need for ACP to be personalized, feasible, and relevant at moments suitable for the individual patient [21]. The existing knowledge gaps regarding ACP initiation, optimal content, and impact are emphasized due to the fragmentation of available evidence and the absence of a holistic evaluative approach [41].

This study contributes to addressing these gaps by shedding light on the patient experience at home and the central role of the patient-HCP relationship in the ACP process which contributes to enhance EoL decision-making especially around preferred place of care and death.

### Implications and future work

The implementation of ACP in primary healthcare involves more than the creation of plans; it requires proactive and ongoing communication among individuals, their loved ones, and HCPs. Aligning with the recommendations of Zwakman et al. [21], a more personalized ACP approach, tailored to the individual patient’s needs, concerns, and coping strategies, is crucial. This personalized approach enhances the effectiveness of ACP interventions and fosters patient-centred care.

Given cancer patients’ preference to stay at home as long as possible, integrating ACP into primary healthcare becomes essential to ensure long-term relationships and continuity of care. Training programs and practical tools within defined ACP interventions could assist HCPs in supporting collaboration among patients, their families, and the healthcare team. This collaborative approach is particularly relevant in primary healthcare settings, where the patient-HCP relationship plays a central role. Efforts are underway to establish ‘days at home’ as a patient-centred outcome in cancer care, serving as a valuable research and policy tool [42].

To advance the field of ACP in primary healthcare, there is a need for higher-quality studies and innovative ACP interventions. These efforts can contribute to the development of effective ACP programs, address existing research gaps, and enhance the overall quality of EoL care for patients with serious illnesses.

### Appraisal of methods

This study adhered to the COREQ guidelines for reporting qualitative research [43]. We employed variation sampling to ensure a diverse range of perspectives and experiences relevant to our research question. Working closely with HCPs in primary healthcare, we prioritized the well-being and dignity of the patients during the recruitment process. After conducting 12 interviews, we believed that we had gathered a sufficient amount of data to effectively address our research question. Additional data collection was deemed unlikely to yield substantially new or different insights. However, the selection of the sample size was predicated on pragmatic considerations, employing a variation sampling strategy. A sample size of 12 participants might be deemed insufficient for achieving data saturation. Saturation might be difficult to assess when it has been reached and there are almost always pragmatic limits on how large or long a study can be [44]. While community norms and prior research can offer valuable guidelines for estimating sample sizes [45], it is imperative not to unquestioningly adopt these norms. Code saturation may indicate when researchers have “heard it all,” but meaning saturation is needed to “understand it all” [46]. The small sample size may limit the potential for generalisation in the current study. We could not get any feedback on the findings from the participants because of their limited lifetime.

The first two interviews were conducted of two persons. The participants could potentially feel uncomfortable if a power imbalance is perceived when meeting two interviewers, and the opportunity for rich data collection could be lost [47]. In these two interviews, we designated roles as interviewer and observer to ensure that the patient’s interaction was primarily directed toward the interviewer.

Thematic analysis was chosen to explore participants’ views and opinions as subjective experiences, providing a rich and detailed account of the data through constant movement back and forth between the entire dataset. The use of a reflexivity journal made our coding and analysis process transparent, documenting how codes and themes supported the findings. Themes were interpreted and understood within a broader context.

To enhance coding reliability, two coders independently coded the same data with three sessions for one coder (CM) and six sessions for another (BD). Regular meetings and discussions among coders and the other researchers further improved coding reliability through clarifications and refining the coding process collaboratively.

The research team comprised individuals with diverse professional backgrounds and scientific experiences, including four physicians, two nurses, and three researchers with backgrounds in medical and natural sciences, encompassing both qualitative and quantitative research. Two authors, BD and AK, brought prior knowledge and experience related to ACP conversations and palliative plans in our region. However, it is important to mention that the two main coders (BD and CM) were not very experienced in qualitative research.

In three interviews, the next of kin was present but not involved with direct questions. Participants’ responses in these cases might have been influenced by the next of kin’s presence, particularly regarding preferences for EoL preparations and desires to remain at home for as long as possible. However, 10 of 12 participants expressed that such decisions couldn’t be made independently without consulting their next of kin.

## Conclusion

Preserving normality at home, coupled with the desire to remain at home for as long as possible, emerges as a significant goal for advanced cancer patients. However, achieving this goal is contingent on physical well-being and the support provided by family caregivers, with uncertainty about the extent of burden patients can impose on others.

Participants expressed a sense of reliance on HCPs for ongoing medical and emotional support. The foundation of trust and a sense of certainty was built and maintained through understandable and compassionate communication over time. In addition to aspects like autonomy and the exercise of control, the ACP process seems to be deeply rooted in personal relationships with HCPs, emerging as a major outcome resulting from discussions about future challenges and EoL considerations.

## Data Availability

No datasets were generated or analysed during the current study.

## Abbreviations

ACP: Advance care planning
EoL: End-of-Life
GP: General Practitioner
HCP: Health Care Personnel
PC: Palliative care
